# The Forkhead Transcription Factor FOXP2 Is Required for Regulation of p21^WAF1/CIP1^ in 143B Osteosarcoma Cell Growth Arrest

**DOI:** 10.1371/journal.pone.0128513

**Published:** 2015-06-02

**Authors:** Duncan M. Gascoyne, Hayley Spearman, Linden Lyne, Rathi Puliyadi, Marta Perez-Alcantara, Les Coulton, Simon E. Fisher, Peter I. Croucher, Alison H. Banham

**Affiliations:** 1 Nuffield Division of Clinical Laboratory Sciences, Radcliffe Department of Medicine, Oxford University, Oxford, OX3 9DU United Kingdom; 2 Wellcome Trust Centre for Human Genetics, Oxford, OX3 7BN United Kingdom; 3 Academic Unit of Bone Biology, Dept of Human Metabolism, University of Sheffield, Sheffield, S10 2RX United Kingdom; 4 Language and Genetics Department, Max Planck Institute for Psycholinguistics, and Donders Institute for Brain, Cognition and Behaviour, Nijmegen, The Netherlands; 5 Garvan Institute of Medical Research, Sydney, Australia; Institut de Génomique Fonctionnelle de Lyon, FRANCE

## Abstract

Mutations of the forkhead transcription factor *FOXP2* gene have been implicated in inherited speech-and-language disorders, and specific Foxp2 expression patterns in neuronal populations and neuronal phenotypes arising from *Foxp2* disruption have been described. However, molecular functions of FOXP2 are not completely understood. Here we report a requirement for FOXP2 in growth arrest of the osteosarcoma cell line 143B. We observed endogenous expression of this transcription factor both transiently in normally developing murine osteoblasts and constitutively in human SAOS-2 osteosarcoma cells blocked in early osteoblast development. Critically, we demonstrate that in 143B osteosarcoma cells with minimal endogenous expression, FOXP2 induced by growth arrest is required for up-regulation of *p21^WAF1/CIP1^*. Upon growth factor withdrawal, FOXP2 induction occurs rapidly and precedes *p21^WAF1/CIP1^* activation. Additionally, FOXP2 expression could be induced by MAPK pathway inhibition in growth-arrested 143B cells, but not in traditional cell line models of osteoblast differentiation (MG-63, C2C12, MC3T3-E1). Our data are consistent with a model in which transient upregulation of Foxp2 in pre-osteoblast mesenchymal cells regulates a p21-dependent growth arrest checkpoint, which may have implications for normal mesenchymal and osteosarcoma biology.

## Introduction

The FOXP2 forkhead transcription factor was identified in 2001 from independent studies mapping mutations associated with human inherited speech-and-language disorder and using homology screening to identify novel forkhead proteins in the mouse lung.[1, 2] FOXP2 shares characteristics with other members of the FOXP subfamily, including a C-terminal winged helix forkhead DNA binding domain and is proposed to function predominantly as a transcriptional repressor.[1, 3, 4] Consistent with neuro-developmental deficits in humans carrying *FOXP2* mutations, this transcription factor is expressed in multiple specific neuronal populations in several species.[5–8] Importantly, Foxp2 expression is not neuronally restricted, having been observed also in normal developing lung epithelium, mesodermal layer of intestine, and cardiac tissues.[1] The related FOXP1 factor is widely expressed in normal and malignant cells[9] and has critical roles during normal development being essential for murine B-cell production.[10] Unlike FOXP1, FOXP2 expression in normal haematopoietic cells appears minimal, although we have identified its frequent expression in malignant myeloma cells (B-cells terminally differentiated into plasma cells) that generally lack FOXP1 expression.[11, 12] FOXP factors have been linked to regulation of the cell cycle via various mechanisms[13, 14] although not thus far to the cyclin-dependent kinase inhibitor *p21* ^*WAF1/CIP1*^, a widely-characterised p53 target gene.[15]

To clarify the mechanisms by which FOXP2 contributes to acquisition of intact speech and language and other developmental functions, multiple research avenues have been pursued. These include generation of animal models with reduced and/or mutated Foxp2 protein expression,[16] the introduction of human evolutionary changes into the mouse orthologue[17] and identification of FOXP2 targets in SH-SY5Y human neuroblastoma and primary neuronal cells.[3, 4, 18–20] Such studies have established important biological roles for Foxp2 in lung and neuronal development, including vocalisation, acquisition of motor-skills, auditory processing, and learning of auditory-motor associations.[16, 17, 21–26] FOXP2 has thereby been implicated as a primary controller of multiple diverse cellular pathways, including adhesion, neurite outgrowth, and neural plasticity.

Given a potential role for FOXP1 in development of bone osteoclasts[27] and expression of FOXP2 in bone-resident myeloma cells (in which the bone niche has an essential role in disease pathogenesis and resistance to therapy),[11] we considered whether FOXP2 might play an important role in bone biology. In accordance with this hypothesis we demonstrate that FOXP2 is required for regulation of *p21* ^*WAF1/CIP1*^ and growth-factor deprivation induced growth arrest of pre-osteoblast type 143B osteosarcoma cells.

## Materials and Methods

### Culture of human osteosarcoma cells and normal human osteoblasts

Human osteosarcoma cell lines were obtained from ATCC and cultured in either DMEM supplemented with 2mM L-glutamine and 10% heat-inactivated FBS (143B), McCoy’s 5A supplemented similarly (U2-OS), MEM supplemented similarly plus 1x non-essential amino acids (MG-63) or McCoy’s 5A supplemented with 2mM L-glutamine and 15% heat-inactivated FBS (SAOS-2, cultured for <20 passages). Addition of 10^-7^M Vitamin D3 (Sigma, Gillingham UK) and 10ng/ml TGF-β1 (Peprotech, London UK) to normal growth media was used to differentiate MG-63.[28] Normal human osteoblasts (NHOst) from Lonza (Slough, UK) were cultured as per supplier instructions. 5T33MM and JJN-3 myeloma cell line controls were cultured in MEM supplemented with 2mM L-glutamine, 1mM sodium pyruvate, 2x non-essential amino acids, 50μg/ml 2-mercaptoethanol and 10% heat-inactivated FBS or RPMI supplemented with 10% heat-inactivated FBS respectively. All media were supplied by Life Technologies, Paisley UK)

### Pathway inhibitor experiments

Growing 143B cells were split in parallel to confluency and sub-confluency and four hours later treated with pathway inhibitors or DMSO vehicle as follows, prior to harvesting for transcript analysis 24hr later: PD-98059 MAPK pathway inhibitor at 50 μM, LY-294002 PI3K pathway inhibitor at 50 μM, IKK pathway inhibitor VII at 1 μM, Bay 117082 NF-κB pathway inhibitor at 1 μM, and DBZ Notch signaling inhibitor at 1 μM. All inhibitors were supplied by Calbiochem (via Millipore, Watford UK) and resuspended in DMSO.

### Alkaline phosphatase and MTS assays

Alkaline phosphatase activity in 10μg of whole cell lysate was determined by addition of PNPP substrate (ThermoFisher Scientific, Loughborough UK) and measurement of absorbance at 405nM. Total viable cell number change was determined by plating cells at 1, 2 or 5 x 10^3^ per well in duplicate 96-well plates, addition of MTS reagent (Promega, Southampton UK) at 24hr or 48hr time points and calculation of 490nM absorbance differences over time (following 630nM background correction).

### RNA isolation, cDNA preparation and real-time PCR

Total RNA was isolated by either Trizol (LifeTechnologies) or SV total RNA isolation method (Qiagen, Manchester UK) and 1μg converted to cDNA using Superscript III enzyme (LifeTechnologies). Routinely 1/25^th^ of each cDNA reaction was used per real-time PCR reaction performed in Universal MasterMix II (LifeTechnologies). Taqman assays (all FAM-labelled) were as follows: Hs_01047977_m1 (*RUNX2*), Hs_00541729_m1 (*SP7/Osterix*), Hs_00173720_m1 (*IBSP*), Hs_00212860_m1 (*FOXP1*), Hs_00362817 (*FOXP2*), Hs_00405889_m1 (*FOXP4*), Hs_00355782_m1 (*CDKN1A*/*p21*), Hs_01597588_m1 (*CDKN1B*/*p27*), Hs_00985639_m1 (*IL6*), Mm_00475030_m1 (*Foxp2*) and Mm_00446968_m1 (*Hprt*). VIC-labelled control probes for human samples were 4319413E (*18S*), 4326322E (*TBP*). Human *FOXP2* transcript levels were verified in several experiments using probe Hs_00362818_m1. Normalisation was routinely performed using both *18S* and *TBP* to confirm target gene effects (*18S* data shown).

### Protein extract preparation and immunoblot analyses

Whole cell extracts from cultured cells were prepared in 10mM HEPES pH7.9, 400mM NaCl, 0.1mM EDTA, 5% glycerol and protease inhibitors after snap freezing, and quantitated by BCA assay (ThermoFisher Scientific). Nuclear extracts from fresh cultures were prepared using an extraction kit (Affymetrix, High Wycombe UK). Protein was extracted from murine bone by Trizol method. 10–40μg of lysate was subjected to immunoblot analysis by standard techniques after 12% SDS-PAGE using primary antibodies as per S1 Table.

### Chromatin immunoprecipitation analyses

Chromatin immunoprecipitation using anti-FOXP2 73A/8, (MABE415 Merck Millipore, Watford UK) and control MR12 antibodies was performed using standard techniques. Briefly, chromatin was prepared from approximately 3 x 10^7^ confluent or serum-starved 143B cells by formaldehyde cross-linking, lysis in SDS lysis buffer (1% SDS, 10 mM EDTA, 50 mM Tris-HCL pH8.0) and 10 cycles of sonication. After clearing of the lysate by centrifugation, and dilution 1:5 into dilution buffer (0.01% SDS, 1% Triton X-100, 2 mM EDTA, 150 mM NaCl, 20 mM Tris-HCl pH 8.0), overnight incubations with 5μg antibody were performed. Immune complexes were collected on Protein A/G-plus agarose (Santa Cruz, Heidelberg, Germany), washed and then eluted into 1% SDS, 0.1M NaHCO_3_. Cross-links were reversed at 65°C, RNAse and proteinase K digestion performed, samples phenol-chloroform extracted and DNA purified into 100 μl T.E. 1 μl template was used for 30 cycles of PCR amplification with specific primers using GoTaq DNA polymerase (Promega). Amplification from a 1% input sample was performed in each case for comparison.

### Flow cytometric analysis of cell cycle distribution

Cell cycle status by distribution of DNA content was determined by flow cytometric analysis (FACScalibur, BD Biosciences, Oxford UK) after fixation in 70% EtOH and resuspension in phosphate-buffered saline containing 0.1% sodium citrate, 0.1% Triton X-100 and 50μg/ml propidium iodide. Data were analysed using FlowJo software.

### siRNA transfection

Human osteosarcoma cells were plated in 6-well dishes at 2 x 10^5^ per well and transfected with Stealth siRNAs, either low GC control duplex 1 (siLGC), *FOXP2* siRNA duplex 1 (P2si#1, targeted sequence 5′-GAC-AGG-CAG-TTA-ACA-CTT-AAT-3′), or *FOXP2* siRNA duplex 2 (P2si#2, targeted sequence 5′-GCG-ACA-GAG-ACA-ATA-AGC-AAC-AGT-T-3′), using RNAiMax reagent (all LifeTechnologies). In some experiments additional control LGC duplexes were used to verify effects.

### Immunohistochemistry of Foxp2 expression

Embryos were formalin fixed, sectioned, and paraffin-embedded slides dewaxed and antigen retrieved by microwaving in 50mM Tris, 2mM EDTA pH9.0, with anti-FOXP2 73A/8 or MR12 antibody (1:1000) subsequently applied overnight at 4°C and the NovoLink polymer system used for detection (LeicaBiosystems, Milton Keynes UK).

### Statistical testing

Statistical testing for significance (indicated where *p*≤0.05 by asterisk) in all experiments was performed by Student’s t-test.

## Results

### Foxp2 is the Foxp factor most highly expressed in murine bone

We have previously observed FOXP2 expression in multiple myeloma,[11] a plasma cell malignancy that is known to interact with the bone microenvironment and cause devastating lytic bone disease in patients.[29] The osteoblast transcription factor RUNX2 is also expressed in myeloma[30] and we hypothesised that similarly FOXP2 might play a role in normal bone biology.

**Fig 1:**
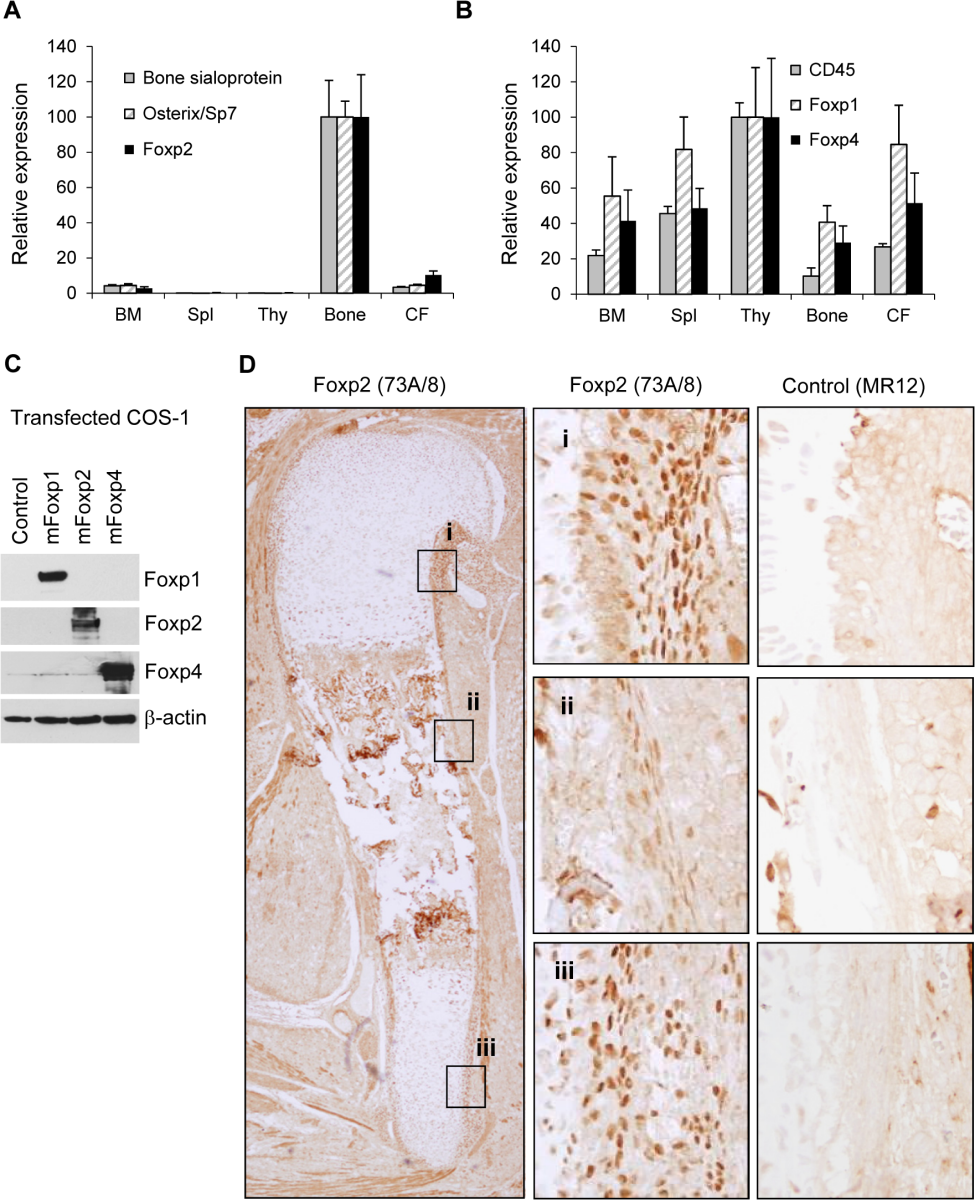
Foxp2/FOXP2 expression in murine bone and association with reduced growth of osteosarcoma cell lines. (A,B) Real-time PCR analysis of gene expression in primary murine tissues from 12-week old male C57Bl/6 mice using SyBr-green, specifically whole bone marrow (BM), spleen (Spl), thymus (Thy), whole long bones after flushing and 2 rounds of collagenase digestion (Bone) and bone-associated cells from the collagenase fraction (CF). Data represent mean +/- SD of three mice. While *Foxp1* and *Foxp4* are most expressed in *CD45*+ haematopoietic tissues, *Foxp2* expression is highest in bone as are the established osteoblast genes *Ibsp* and *Sp7*. Expression normalised to *Hprt*, expressed relative to highest sample (100%); (C) Immunoblot analysis of COS-1 fibroblast-like cells transiently transfected with CMV-driven mammalian expression plasmids containing murine full-length Foxp cDNAs or pCDNA4 empty vector (control) as indicated top. All antibodies exhibited specificity for appropriate ectopically-expressed proteins, low level endogenous FOXP4 expression was detectable in all lysates; (D) Immunohistochemical detection of Foxp2 protein in murine E17.5 long bone, detail (middle panels) showing variation in Foxp2 positivity along the periosteum, staining with the anti-rabbit murine monoclonal antibody MR12 was performed on serial sections as negative control (same regions, right panels)

Preliminary data-mining analysis of *Foxp2* gene expression patterns in normal tissues using www.biogps.org identified potentially high-level *Foxp2* transcript expression in osteoblasts from multiple probesets 1422014_at, 1438231_at, 1438232_at and 1440108_at (data not shown). We confirmed this expression profile of *Foxp2* transcripts in murine bone (Fig 1A), and found it to contrast with expressions of *Foxp1* and *Foxp4* (Fig 1B). To measure Foxp2 expression in normal osteoblast development we studied normal E17.5 long bone after validating our anti-FOXP2 73A/8 monoclonal antibody for Foxp2 reactivity and specificity against the widely expressed murine Foxp proteins (Fig 1C). We found strong nuclear expression of Foxp2 in periosteal cells at the bone collar (Fig 1D panels i and iii), but not in more mature osteoblasts located towards the centre of the periosteum (Fig 1D panel ii). Consistent with a recent report[31] weak Foxp2 expression was also present in proliferating chondrocytes (S1 Fig). Combined, these data suggest FOXP2 to be the most important FOXP factor for bone biology.

### FOXP2 is expressed in SAOS-2 cells and induced by growth arrest in MG-63

**Fig 2:**
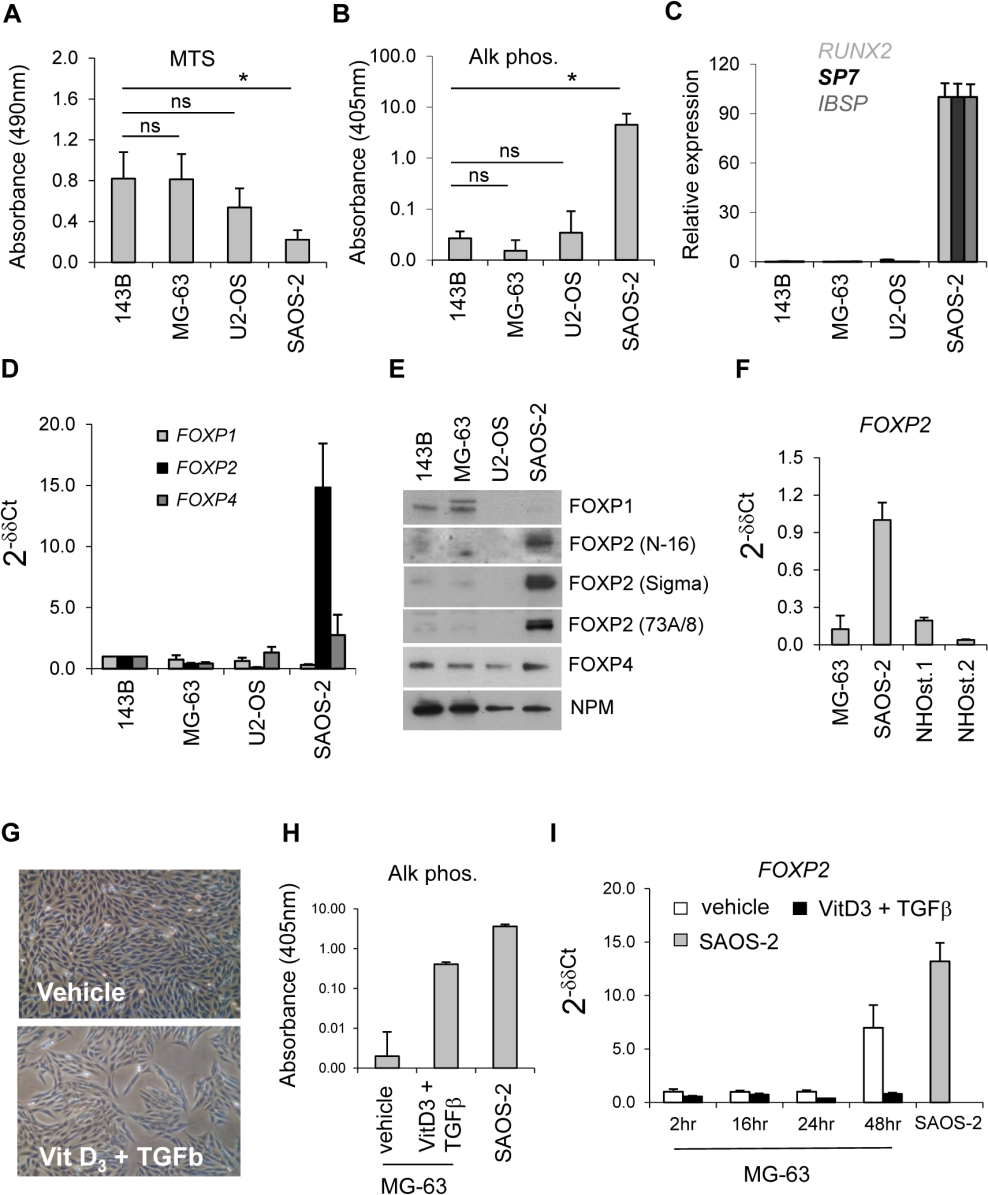
FOXP2 is expressed in SAOS-2 cells and induced by growth arrest in MG-63. (A) Total viable cell number determined by MTS assay (absorbance at 490nm) in cell lines 143B, MG-63, U2-OS and SAOS-2 growing exponentially *in vitro*, after plating at equal cell numbers *N* = 3 ± SD; (B) Alkaline phosphatase activity by PNPP assay (absorbance at 405nm) in whole cell lysates, *N* = 3 ± SD; (C) Real-time PCR analyses of gene expressions as indicated, expression shown as percentage relative to highest (100%) *N* = 3 ± SD; (D) Real-time PCR analyses of *FOXP* expression as indicated, expressed as 2^-δδCT^, relative to 143B, *N* = 3 ± SD; (E) Immunoblot analyses of nuclear extracts from exponentially growing cell lines, including nucleophosmin (NPM) as a loading and transfer control, representative of two experiments; (F) Real-time PCR analysis of human *FOXP2* expression in NHOst cultures from two individuals, expressed as 2^-δδCT^, relative to expression in SAOS-2 sample, parallel immunoblot analysis did not detect FOXP2 in NHOst cultures (not shown); (G) Phase contrast images of MG-63 cultures plated subconfluently and cultured for 48hr in presence of vehicle alone or Vitamin D_3_ plus TGF-β to induce osteoblast differentiation; (H) Alkaline phosphatase activity in lysates from cells in *H* and SAOS-2 positive control; (I) Real-time PCR analyses of *FOXP2* expression in MG-63, relative to 2hr vehicle sample, all MG-63 differentiation data representative of two experiments.

To further investigate FOXP2 expression in cells with osteoblast characteristics *in vitro* we examined established murine MC3T3-E1 and C2C12 systems for Foxp2 expression. However, expression of *Foxp2* was extremely low and was not regulated significantly by the differentiation stimulus in either system (data not shown). Thus a series of human osteosarcoma cell lines whose differentiation are blocked during pre-osteoblast mesenchymal development was investigated. Of these, SAOS-2 exhibit the slowest growth and significant alkaline phosphatase activity (key characteristics of osteoblasts), along with expression of hallmark osteo-lineage genes *RUNX2* (bone-specific isoform,[32]) *Osterix*/*SP7*, and integrin-binding sialoprotein *IBSP* (Fig 2A–2C). These data indicate that SAOS-2 exhibit a committed osteoblast phenotype being more mature than 143B, MG-63 or U2-OS. Interestingly, this state is associated with robust FOXP2 expression at both the transcript and protein levels (Fig 2D and 2E). Given the paucity of literature on FOXP expression in the osteoblast lineage, FOXP2 expression was confirmed using multiple antibodies (Fig 2E) and additional real-time PCR assays (amplifying Exons 14–15 and Exons 16–17 respectively, data not shown). FOXP4 levels across the panel were relatively constant while FOXP1 expression was lowest in U2-OS and SAOS-2. Consistent with data from the embryonic murine bone, normal mature human osteoblasts from multiple donors did not express significant *FOXP2* transcripts or protein (Fig 2F and data not shown)

To investigate whether FOXP2 expression might define a more committed stage of osteoblast development we used an established protocol[28] to promote differentiation of MG-63 cells, thereby inducing growth arrest and alkaline phosphatase expression, while monitoring *FOXP2* expression (Fig 2G–2I). The combined Vitamin D3 and TGF-β treatment, which arrested cell growth at sub-confluence and induced alkaline phosphatase activity (Fig 2G and 2H), did not induce *FOXP2* (Fig 2I). Interestingly, the vehicle-treated cells, which became growth arrested by confluence at the end of the experiment showed an approximately 7-fold significant increase in *FOXP2* expression after 48h (Fig 2I).

### Confluent growth arrest is associated with increased FOXP2 expression

**Fig 3:**
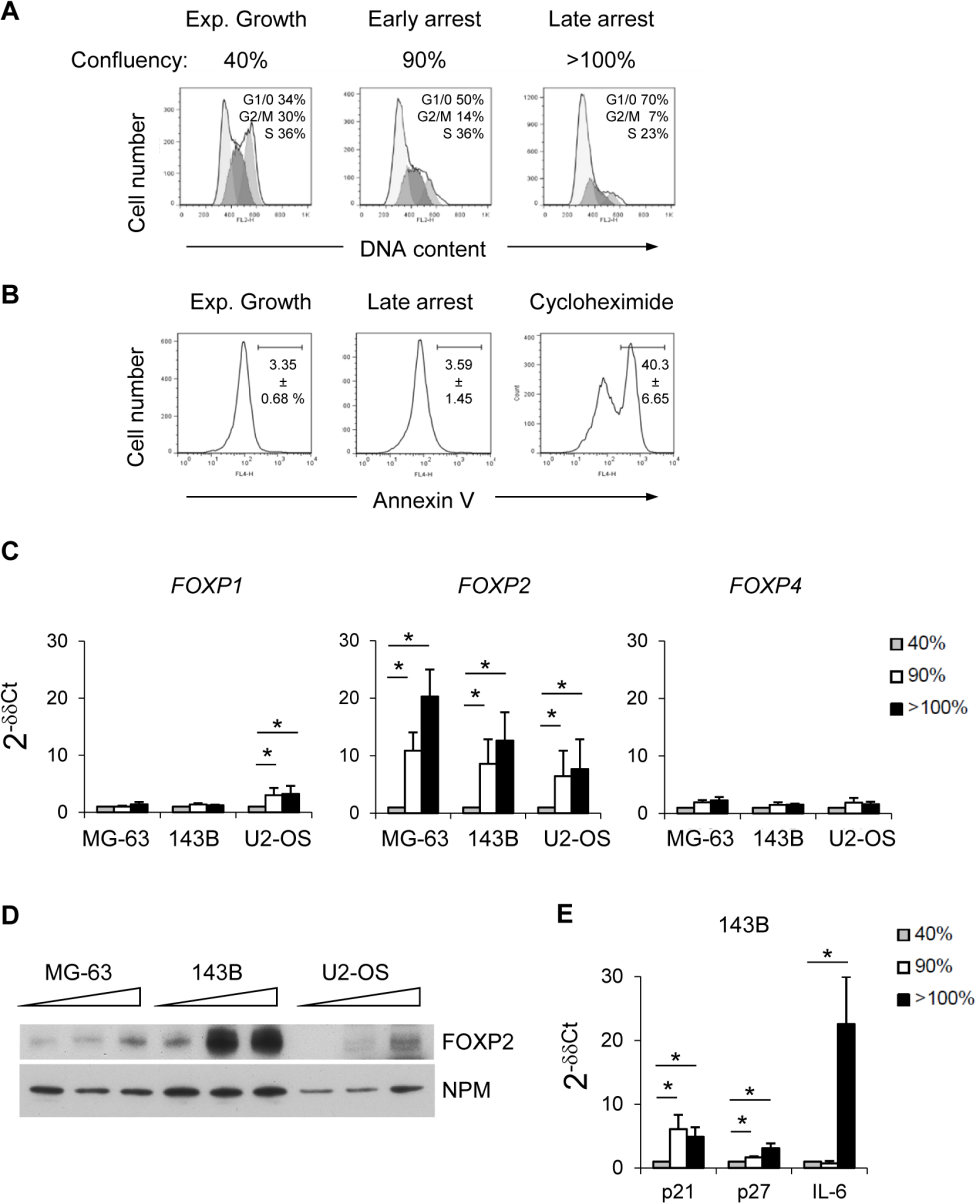
Confluent growth arrest is associated with increased FOXP2 expression. (A) Cell cycle analysis of 143B subjected to increasing confluence (approximate percentage confluence indicated top), numbers within plots from left to right indicate percentage of cells in G1/G0, S, and G2/M phase respectively, representative of three experiments; (B) Quantitation of apoptotic cell death in 143B cell populations by flow cytometric analysis of Annexin V positivity, in cultures either exponentially growing (Exp. Growth), subjected to overnight culture with 20μg/ml cyclohexmide as a positive control that induces apoptosis (cycloheximide), or subjected to 4 days growth arrest at confluence (late arrest, as per *A*). Numbers represent mean % annexin positive ± SD from three experiments; (C) Real-time PCR analyses of *FOXP* expression in MG-63, 143B and U2-OS cultured to increasing confluence, expressed as 2^-δβCT^, relative to growing culture, *N* = 3 ± SD; (D) Immunoblot analyses of nuclear extracts from cells cultured as in *C*, including nucleophosmin (NPM) as a loading and transfer control, representative of two experiments, (E) Real-time PCR analyses of *p21*, *p27* and *IL-6* expression in 143B cultured to increasing confluence, expressed as 2^-δδCT^, relative to growing culture, *N* = 3 ± SD.

To determine the wider applicability of confluent growth arrest (rather than terminal differentiation) in FOXP2 induction, cell lines with minimal basal FOXP2 expression (MG-63, 143B, U2-OS) were cultured to confluency (Fig 3). Confluence was associated with growth arrest and minimal apoptosis (143B data shown as example, Fig 3A and 3B), even after 4 days and significant, up to 20-fold, induction of *FOXP2* transcripts in all cell lines (*p*<0.05 for both early and late arrest states Fig 3C). Expression of other *FOXP* family members (*FOXP1* and *FOXP4)* remained relatively unaffected (Fig 3C), the only significant effect being weak *FOXP1* induction in both U2-OS arrest samples (*p*<0.05). Parallel immunoblotting analyses validated robust FOXP2 protein increases by confluence in 143B cells (Fig 3D), although not in MG-63 and U2-OS cells where background *FOXP2* transcription is low (Fig 2D) Inhibition of CDK kinase activity during growth arrest is critical for reduction of pRb levels and/or its phosphorylation and resulting decreases in E2F transcription factor availability. This CDK inhibition is dependent upon several cyclin-dependent kinase inhibitors (CKIs) including CDKN1A/p21^WAF1/CIP1^ and CDKN1B/p27^KIP1^ (for review see [33]). While VitD_3_/TGFβ-dependent MG-63 differentiation occurred in the absence of cell-cycle dependent kinase inhibitor *p21* ^*CIP1/WAF1*^ and *p27* ^*KIP1*^ up-regulation (S2 Fig), induction of full-length FOXP2 in confluent 143B cells was coincident with strongly induced *p21* ^*CIP1/WAF1*^ and more weakly induced *p27* ^*KIP1*^ transcripts (*p*<0.05, Fig 3E).

### Growth arrest-induced FOXP2 transcription is upstream of the cell-cycle machinery

**Fig 4:**
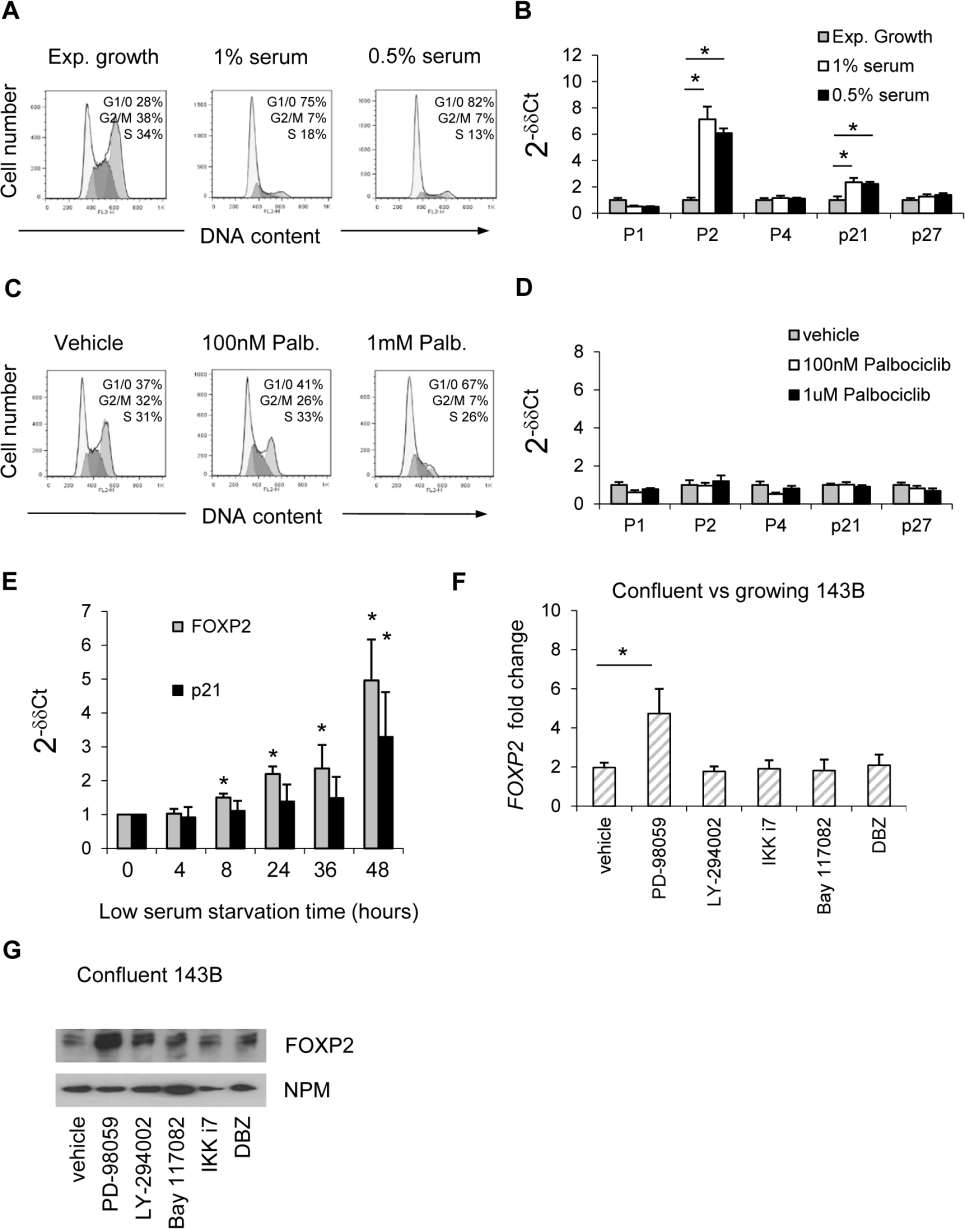
Growth arrest-induced FOXP2 transcription is upstream of the cell cycle machinery. (A, C) Cell cycle analyses of 143B grown for 6 days in reduced serum or 1 day in the CDK4/6 inhibitor Palbociclib 100 nM or 1 μM as indicated; (B, D) Real-time PCR analyses of gene expression in 143B cultured as in *A* and *C*, expressed as 2^-δδCT^, relative to growing or vehicle-treated culture, *N* = 3 ± SD; (E) Real-time PCR analyses of gene expression in 143B reduced serum experiments similar to *A* over a shorter timecourse, *N* = 3 ± SD; (F) Real-time PCR analysis of *FOXP2* expression in 143B cultured at subconfluence or confluence for 24hrs in the presence of inhibitors/vehicle as indicated, (PD-98059 ERK1/2 inhibitor and LY-294002 PI3K inhibitor at 50μM, IKK inhibitor 7, Bay117082 NFκB inhibitor, and DBZ Notch pathway inhibitor at 1μM), expressed as fold change induced by confluence, inhibitors had minimal effect on subconfluent *FOXP2* expression, *N* = 5 ± SD; (G) Immunoblot analysis of nuclear extracts from 143B treated at confluence as in *F*, including nucleophosmin (NPM) as a loading and transfer control.

We considered that induction of FOXP2 by cell confluence might be a consequence either of growth arrest or increased cell-cell interactions as a consequence of cell density (or both). When 143B growth was inhibited at sub-confluence by five days’ serum restriction (Fig 4A), rather than contact inhibition, *FOXP2* induction was maintained (*p*<0.05, Fig 4B), indicating this to be a growth-inhibition rather than cell-cell contact related phenomenon. Low serum treatment was also associated with significant increases in *p21* ^*WAF1/CIP1*^ transcript levels (*p*<0.05, Fig 4B). Growth arrest of 143B in G1/0, induced by the downstream Cyclin-Dependent Kinase (CDK) inhibitor Palbociclib,[34] (Fig 4C) was not associated with *FOXP2* induction or that of *p21* ^*WAF1/CIP1*^ (Fig 4D), indicating *FOXP2* induction to be an upstream primary event rather than secondary to growth arrest. Experiments to determine the temporal relationship between FOXP2 and *p21* ^*WAF1/CIP1*^ induction after serum starvation demonstrated early increases in *FOXP2* transcript levels prior to *p21* ^*CIP1/WAF1*^ induction (Fig 4E). Specifically, *FOXP2* induction was significant at 8, 24, 36 and 48 hr timepoints, while *p21* ^*CIP1/WAF*^ significant only at 48hr. In an attempt to block the FOXP2 induction by confluence, experiments were repeated with the addition of various chemical inhibitors of biological signalling pathways. While no inhibitor blocked *FOXP2* induction, ERK1/2 pathway inhibition using PD-98059 significantly increased FOXP2 induction approximately 2-fold (Fig 4F *p* = 0.03, and Fig 4G), providing further evidence for loss of proliferation signals driving FOXP2 expression.

### FOXP2 induction is required for efficient 143B growth arrest and p21 ^CIP1/WAF1^ upregulation

**Fig 5:**
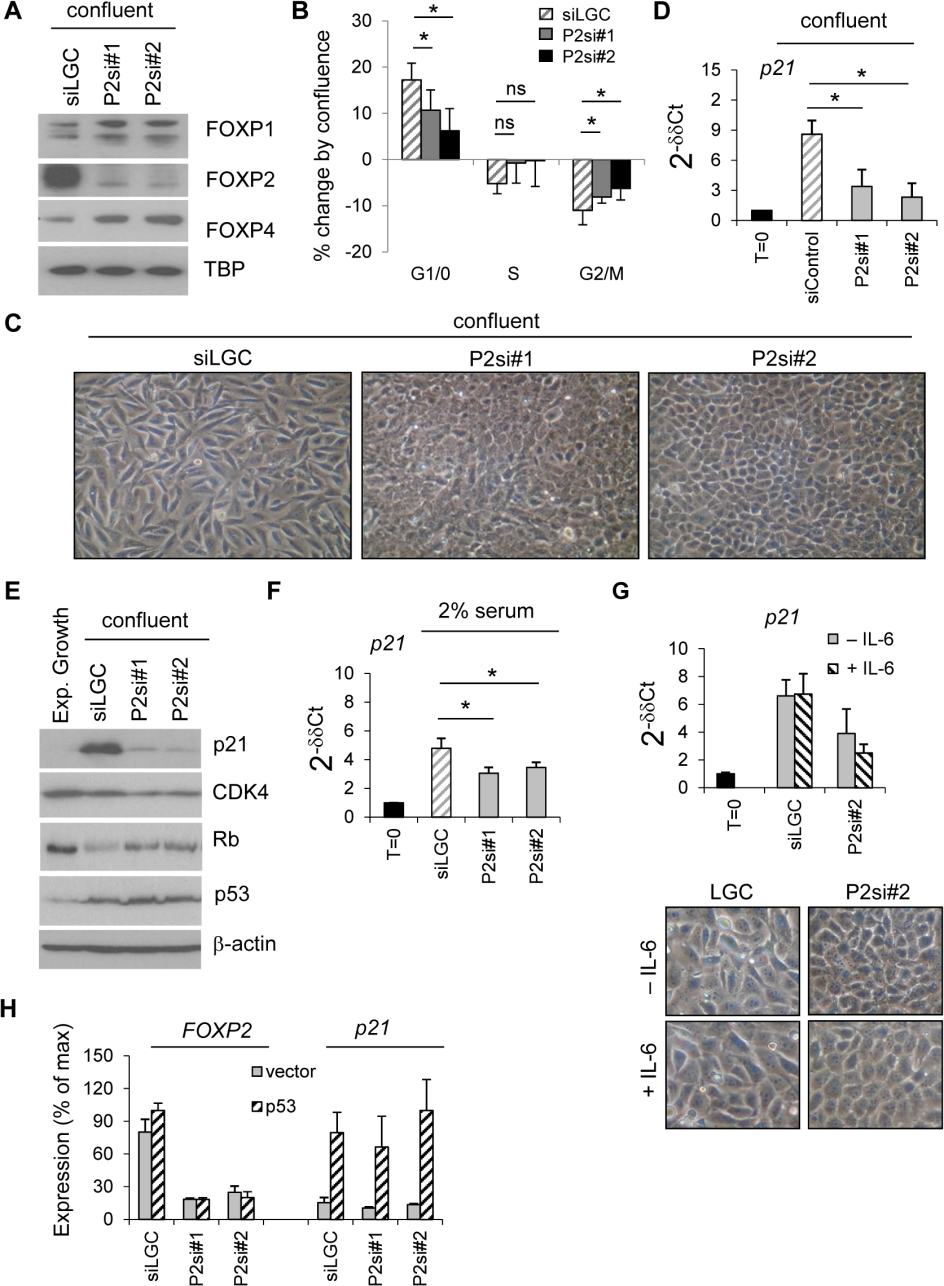
FOXP2 induction is required for efficient 143B growth arrest and p21 ^CIP1/WAF1^ control. (A) Immunoblot analysis of FOXP expression in nuclear extracts from confluent 143B cells 48hr after siRNA transfection, including TATA-binding protein (TBP) as a loading and transfer control, representative of three experiments; (B) Quantitation of changes in cell cycle fractions induced by confluence as per *A*, relative to dividing cells, *N* = 3 ± SD; (C) Phase contrast images of cells as in *A*, to show frequently increased saturation density following FOXP2 depletion;; (D) Real-time PCR analysis of *p21/CDKN1A* expression in 143B cultured as in *A*, expressed as 2^-δδCT^, relative to subconfluent culture (T = 0), control is mean of four different control siRNAs. *N* = at least 4 ± SD; (E) Immunoblot analyses of whole cell lysates from cells cultured as in *A*; (F) Real-time PCR analysis of *p21CDKN1A* expression in cells transfected at subconfluence (T = 0) with siRNAs as shown and harvested still at subconfluence after 48 hr culture in 1% serum, *N* = 3 ± SD; (G) Real-time PCR analysis of *p21CDKN1A* expression in and phase contrast images of cells cultured as in *A*, in the presence of either 10ng/ml hIL-6 (+ IL-6) or PBS/BSA carrier alone (—IL-6); (H) Real-time PCR analyses of cells transfected at subconfluence with p53 cDNA plasmid or empty control plasmid (vector), then treated with siLGC or each FOXP2 siRNA and harvested still at subconfluence after 48hr culture in 10% serum. Expression is shown as percentage relative to highest (100%).

To understand the importance of FOXP2 induction for confluent growth arrest, 143B cells were treated with *FOXP2*-targeting or control siRNAs at sub-confluence and then, after being grown to confluence, cell morphology and cell cycle status were monitored (Fig 5). Efficient and specific targeting of FOXP2 at the protein level was achieved and was associated with some increases in FOXP1 and FOXP4 expression, which might be via a compensatory mechanism (Fig 5A). Under these conditions FOXP2 depletion was associated with reduced growth arrest as determined by significantly reduced changes in G1/0 and G2/M (*p*<0.05 for both siRNAs, Fig 5B) and increased cell density (Fig 5C).

Following both confluent and low-serum growth arrest we observed *CDKN1A*/*p21* to be induced in 143B cells (Figs 3E, 4B and 4E). Timecourse experiments under serum starvation already indicated *FOXP2* induction occurs earlier than *CDKN1A* and critical FOXP2 depletion experiments showed that the majority of *CDKN1A* transcript induction by confluence is FOXP2-dependent (expression after either FOXP2 siRNA significantly lower than control *p*<0.001, Fig 5D). At the protein level, FOXP2 depletion reduced p21 protein induction and consequential total Rb downregulation whilst leaving total p53 and CDK4 levels largely unaltered (Fig 5E, although CDK4 expression in some experiments as shown here was slightly reduced). In addition, *CDKN1A/p21* expression induced by serum deprivation was also partially FOXP2-dependent (both siRNAs *p*≤0.05, Fig 5F).

Mechanistically, in accordance with positive regulation of *p21* by IL-6 in osteoblastic cells[35], also IL-6 expression was induced under confluent conditions (Fig 3E). However, addition of exogenous IL-6 did not rescue *p21* decreases or morphology changes induced by FOXP2 depletion (Fig 5G), indicating control of *p21* by FOXP2 to be independent of IL-6. Given interactions between other FOXP family members and the p53 pathway[36, 37] and induction of the *CDKN1A* transcriptional regulator p53 during growth arrest of 143B (Fig 5E), a specific requirement for FOXP2 in p53-dependent *p21* transcription was examined. Importantly, in subconfluent 143B cells which are *p53* mutant[38, 39] induction of *p21* transcription by ectopic expression of wild-type p53 did not upregulate *FOXP2* and *FOXP2* siRNA, demonstrating that this p21 induction by p53 was independent of the basal FOXP2 expression present in exponentially growing cells (Fig 5H).

In order to understand further how FOXP2 might control *p21*, if not via regulation of IL-6 expression or p53 activity, chromatin immunoprecipitation on lysates from growth arrested 143B cells was performed using control and anti-FOXP2 antibodies and multiple primer pairs proximal to 14 clusters of potential FOXP binding sites within 10Kb upstream or downstream of the *p21/CDKN1A* transcription start site. Despite efficient FOXP2 immunoprecipitation using the 73A/8 monoclonal antibody under ChIP conditions, binding of FOXP2 at specific sites in the *p21* locus was not reliably detected (S3 Fig). These data, together with an absence of *p21*/*CDKN1A* from published lists of direct FOXP2 target genes in other systems,[3, 4, 19] indicate a likely indirect mechanism for FOXP2-dependent control of *p21* transcription.

In summary, our expression and functional data implicate FOXP2 as a transient regulator of growth arrest via indirect control of p21^WAF1/CIP1^ at an early stage of pre-osteoblast mesenchymal development.

## Discussion

Studies over the last fifteen years have established FOXP2 as a molecule important for neurobiology, particularly in the acquisition of speech and language as evidenced by its mutation in families with inherited speech and language disorders.[2, 8] However, expression of FOXP2 is not restricted to neuronal populations, and is important for example also in lung epithelial development in the mouse.[23] Here we have shown that FOXP2 is expressed in developing osteoblasts and have demonstrated that FOXP2 can promote growth arrest and therefore likely control differentiation by regulating expression of the critical cell-cycle inhibitor p21^WAF1/CIP1^.

These findings, together with those of Zhao et al [31] add FOXP2 to the list of established osteoblast and chondrocyte transcription factors such RUNX2,Osterix and SOX9 [40, 41] and it will be interesting in the future to investigate the interplay between FOXP2 and these factors, and understand their potential role in regulation of p21 expression alongside Foxp2. It is potentially noteworthy that *Foxp2* expression in developing embryonic bone may be increased by *Runx2* deletion (GEO profile GDS2185 / 164118_at / Foxp2), with the stage of developmental block in *Runx2* ^-/-^ bone potentially being defined by the induction of *Foxp2* expression. Furthermore the induction of FOXP2 in 143B growth arrest is associated with induction of the chondrocyte transcription factor SOX9 (unpublished data). It is also likely that additional transcription factors interplay with Foxp2 in developing bone, and we note that deficiency of the Lim-domain factor Lhx8, whose expression is most closely correlated with that of Foxp2 in murine tissues (both osteoblast and neuronal expression- www.biogps.org), causes cleft palate and molar deficiencies in mouse models.[42, 43]

We have identified that FOXP2 expression is associated with that of both p21^WAF1/CIP1^ and IL-6, whose importance and interplay in osteoblast lineage cells has been studied previously.[35, 44, 45] However, in contrast to previous findings,[35] our data show that exogenous IL-6 cannot rescue reductions in *p21* ^*WAF1/CIP1*^ expression following FOXP2 depletion. These data indicate that FOXP2-dependent regulation of *p21* ^*WAF1/CIP1*^ in osteoblast lineage cells is independent of IL-6. Given that multiple published FOXP2 ChIP studies have not identified binding to the *CDKN1A* locus, and that also our data do not support FOXP2 binding to a discrete *CDKN1A* region, further studies will be required to identify indirect mechanisms by which FOXP2 controls p21 transcription. One possible intermediate is MEF2D, which has been identified both as a FOXP2 target[3] and recently as a direct regulator of p21/CDKN1A transcription.[46] Given the importance of p21^WAF1/CIP1^ expression in neuronal development (for example, Marques-Torrejon et al[47]), control of p21 by Foxp2 is likely to play an important role in controlling neuronal differentiation. Interestingly, based on data from genome-wide expression profiling experiments, *p21*/*CDKN1A* levels in SH-SY5Y cells ectopically expressing human FOXP2 protein are significantly higher than in SH-SY5Y cells ectopically expressing the chimp orthologue,[18] suggesting the possibility of altered FOXP2-p21 regulation during human evolution. Regarding the RUNX-2-dependent pathway for FOXP function in mesenchymal lineage proposed by Zhao et al[31] our data for active FOXP2 function in 143B cells demonstrate that the bone-specific isoform of RUNX2, which these cells lack, is not a requirement for FOXP2 function.

A recent *in vivo* knockout study[31] has demonstrated that the control of chondrocyte and osteoblast development by Foxp proteins is a complicated process, and it is evident that of the Foxp factors, Foxp2 loss has arguably the greatest disruptive effects on normal chondrocyte and osteoblast biology. We have successfully identified 143B cells as a complimentary tractable *in vitro* model system for studying FOXP2 and *p21* ^*WAF1/CIP1*^ co-regulation in mesenchymal growth arrest, which will enable further studies to more quickly elucidate mechanisms for FOXP2 function. While similar treatment of additional osteosarcoma lines induces *FOXP2* transcripts, upregulation of FOXP2 protein is limited (Fig 3), and confluent growth arrest of the neuroblastoma line SH-SY5Y does not induce *FOXP2* transcripts (data not shown). We speculate that as for RUNX2 in osteosarcoma,[32] the expression and/or function of FOXP2 in malignant cells may be frequently compromised, which may explain the lack of overt growth arrest phenotype and induction of *p21* ^*WAF1/CIP1*^ in SH-SY5Y cells stably engineered to over-express recombinant FOXP2.[3, 4] Alternatively, FOXP2 may require one or more co-operating factors to initiate growth arrest in normal developing populations. This would be consistent with a model in which established specific pro-osteoblast signals (such as BMPs driving RUNX2 but not FOXP2) function synergistically with signals triggered by loss of growth signalling activity (such as FOXP2 after MAPK pathway inhibition). Indeed, important interactions between Foxp3 and Runx family proteins have been demonstrated previously in hematopoietic cells[48] and also between Foxps and Runx2 recently in murine bone.[31]

Our expression studies suggest that in developing murine mesenchymal cells the loss of growth factor signalling upon entry to the periosteum might up-regulate Foxp2. Expression of Foxp2 during both osteoblast and some neuronal development appears to be transient (this manuscript and for example Rousso et al[20]). This is also observed for other family members, for example transient FOXP3 induction in activated human T-cells[49] in contrast to maintained FOXP3 expression in regulatory T-cell populations.[48] Such temporal restriction in specific populations may relate to renewal of (alternate) growth signalling at later stages of development, or negative *FOXP2* autoregulation once growth arrest function has been completed.

Given the importance of the growth arrest gene *p21* ^*WAF1/CIP1*^ as a p53 target[15] and the roles of p53 in osteoblast development and osteosarcoma[50, 51] we considered potential connections between FOXP2 and p53 pathways, as have been demonstrated for the related FOXP1 and FOXP3 proteins.[36, 37] However, thus far the lack of correlation between FOXP2 expression and *p53* status in cell lines (143B mutant, MG-63 null, U2OS wild-type, SAOS-2 null), absence of FOXP2 expression changes upon p53 overexpression, and lack of requirement for FOXP2 in p53-dependent *p21* activation indicate that FOXP2 and p53 function independently to regulate one or more common targets such as p21. Given the recent identification of SOX2 inhibition by p21,[47] our findings provide a potentially novel mechanism for the published inverse relationship of Sox2 with Foxp2 in developing motor neurons.[20]

Our findings have implications not only for normal development but also human disease. Normal osteoblast development is compromised in bone metastases of solid tumours and in bone malignancies such as multiple myeloma, and thus characterisation of FOXP2 growth arrest function in a disease context may identify novel malignant pathways. Furthermore, FOXP2 status in osteosarcoma may provide information regarding the stage of developmental block, with potential clinical significance. Direct connection of FOXP2 to the mutated p53 pathway in osteosarcoma, via a common target p21/CDKN1A, is also likely to have implications for understanding osteosarcoma biology.

## Supporting Information

S1 Table Details of antibodies used in this study. (TIF)

S1 Fig Weak Foxp2 expression in proliferating embryonic murine chondrocytes. Detail of images partly shown in Fig 1, immunohistochemical detection of Foxp2 protein in murine E17.5 long bone, demonstrating weak but significant Foxp2 positivity in proliferating chondrocytes (at the location indicated by arrow). Staining with the anti-rabbit murine monoclonal antibody MR12 was performed on serial sections as negative control.
(TIF)

S2 Fig Growth arrest and differentiation of MG-63 occurs without induction of *p21* and *p27* transcripts. Real-time PCR analyses of *p21* expression (A) and *p27* expression (B) in MG-63, relative to 2hr vehicle sample.
(TIF)

S3 Fig Attempted immunoprecipitation of FOXP2 on the human *p21* ^*WAF/CIP*^ locus. (A) Initially, confluent 143B cell lysates were immunoprecipitated with MR12 or 73A/8 FOXP2 antibodies as indicated, washed as per ChIP protocol and precipitated complexes analysed by immunoblot for presence of FOXP2 protein. (B) Co-precipitated chromatin from similar experiments was used as template for amplification of fragments of the human *p21* ^*WAF1/CIP*^ locus. Site labelled ‘con’ was chosen as a negative control, being (within the 40kb analysed) the greatest distance away from predicted FOXP binding sites. Although some apparent FOXP2 binding at sites 5 to 8 was observed in the experiment shown it could not be replicated in a further five experiments.
(TIF)
