# Supplementary figures and images for: The Forkhead Transcription Factor FOXP2 Is Required for Regulation of p21WAF1/CIP1 in 143B Osteosarcoma Cell Growth Arrest
Source: PLoS One. 2015 Jun 2;10(6):e0128513. doi: 10.1371/journal.pone.0128513 (PMC4452790; doi:10.1371/journal.pone.0128513)

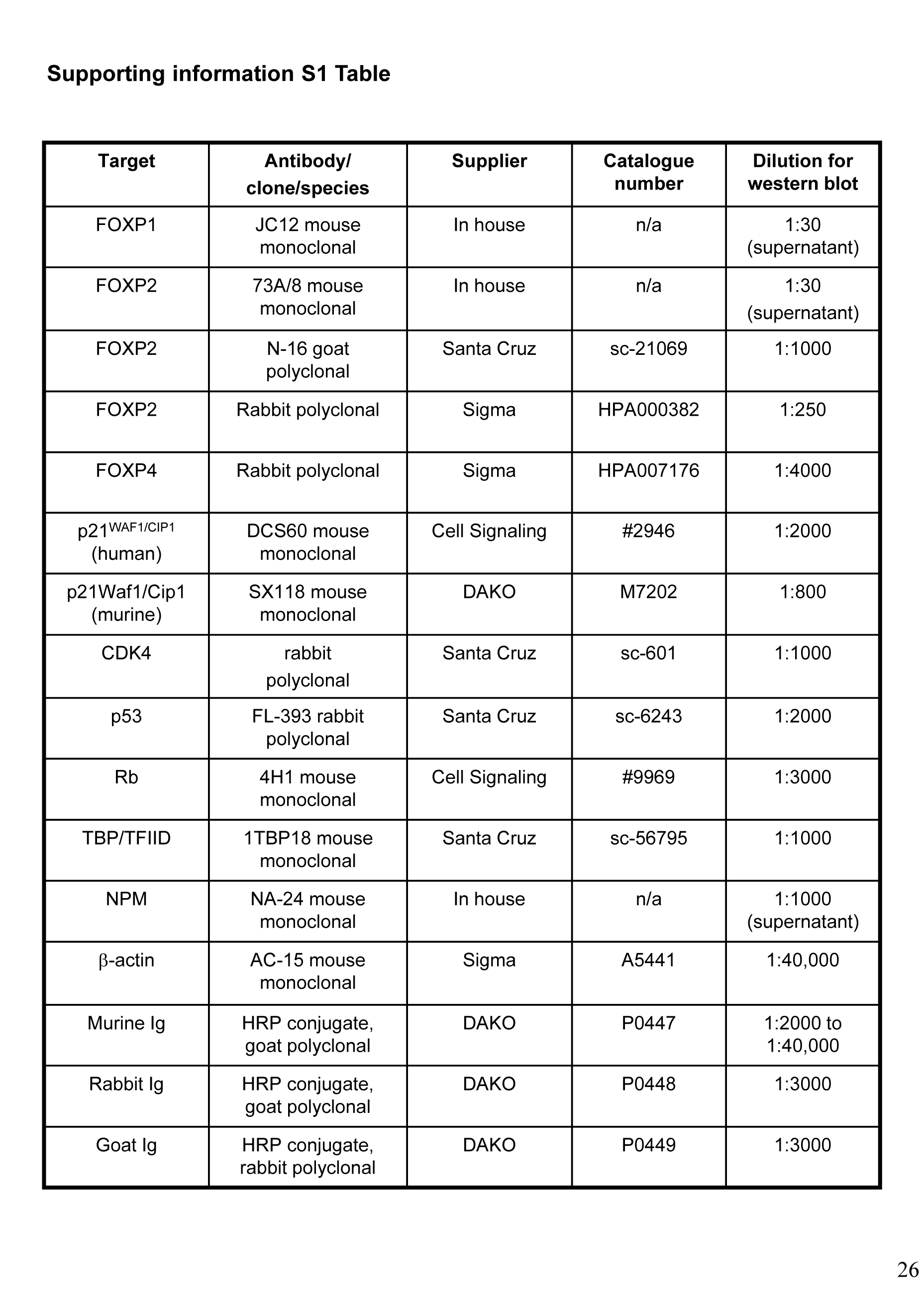

Supplement: S1 Table — (TIF) [file pone.0128513.s001.tif]

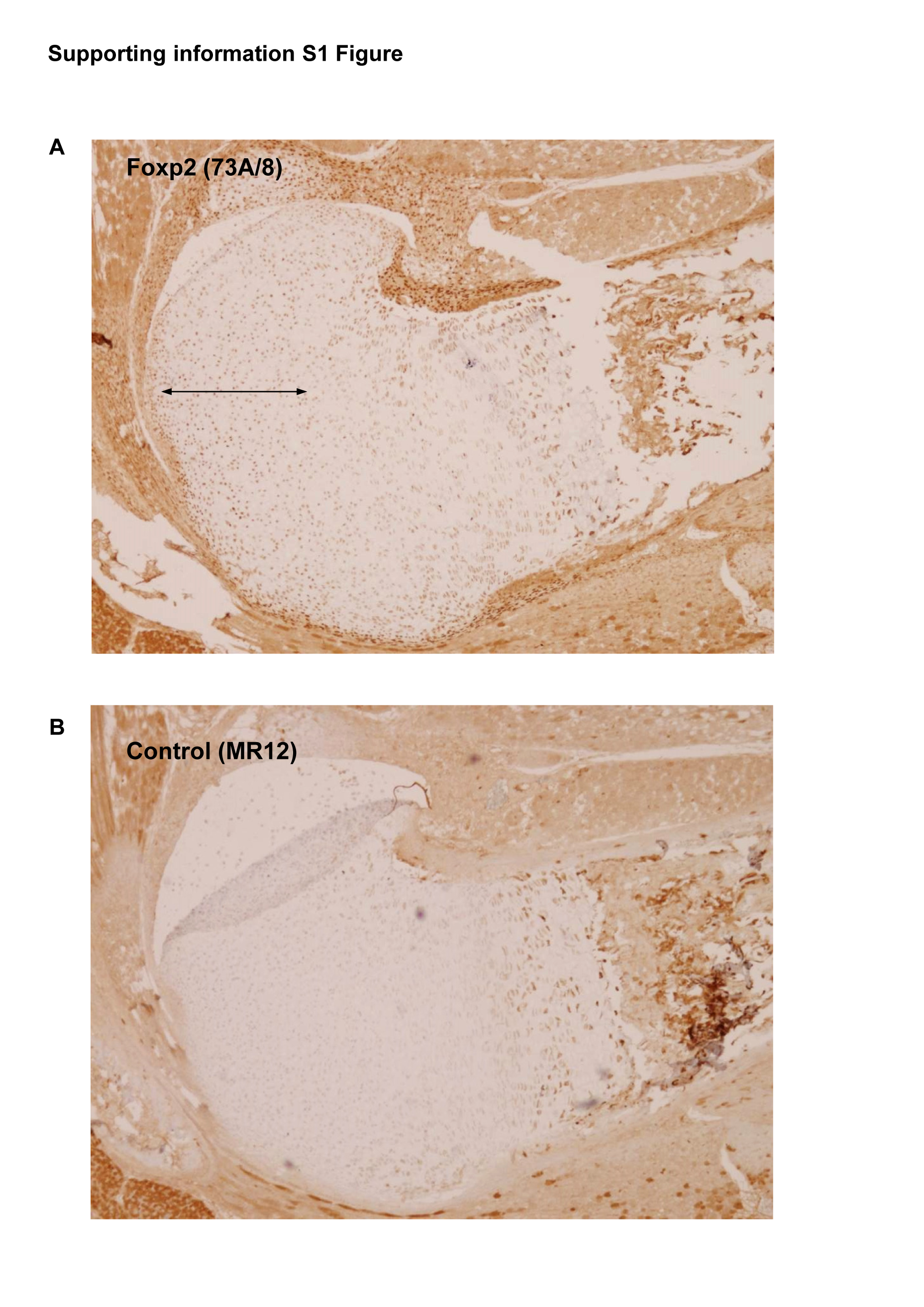

Supplement: S1 Fig — Detail of images partly shown in Fig 1, immunohistochemical detection of Foxp2 protein in murine E17.5 long bone, demonstrating weak but significant Foxp2 positivity in proliferating chondrocytes (at the location indicated by arrow). Staining with the anti-rabbit murine monoclonal antibody MR12 was performed on serial sections as negative control. (TIF) [file pone.0128513.s002.tif]

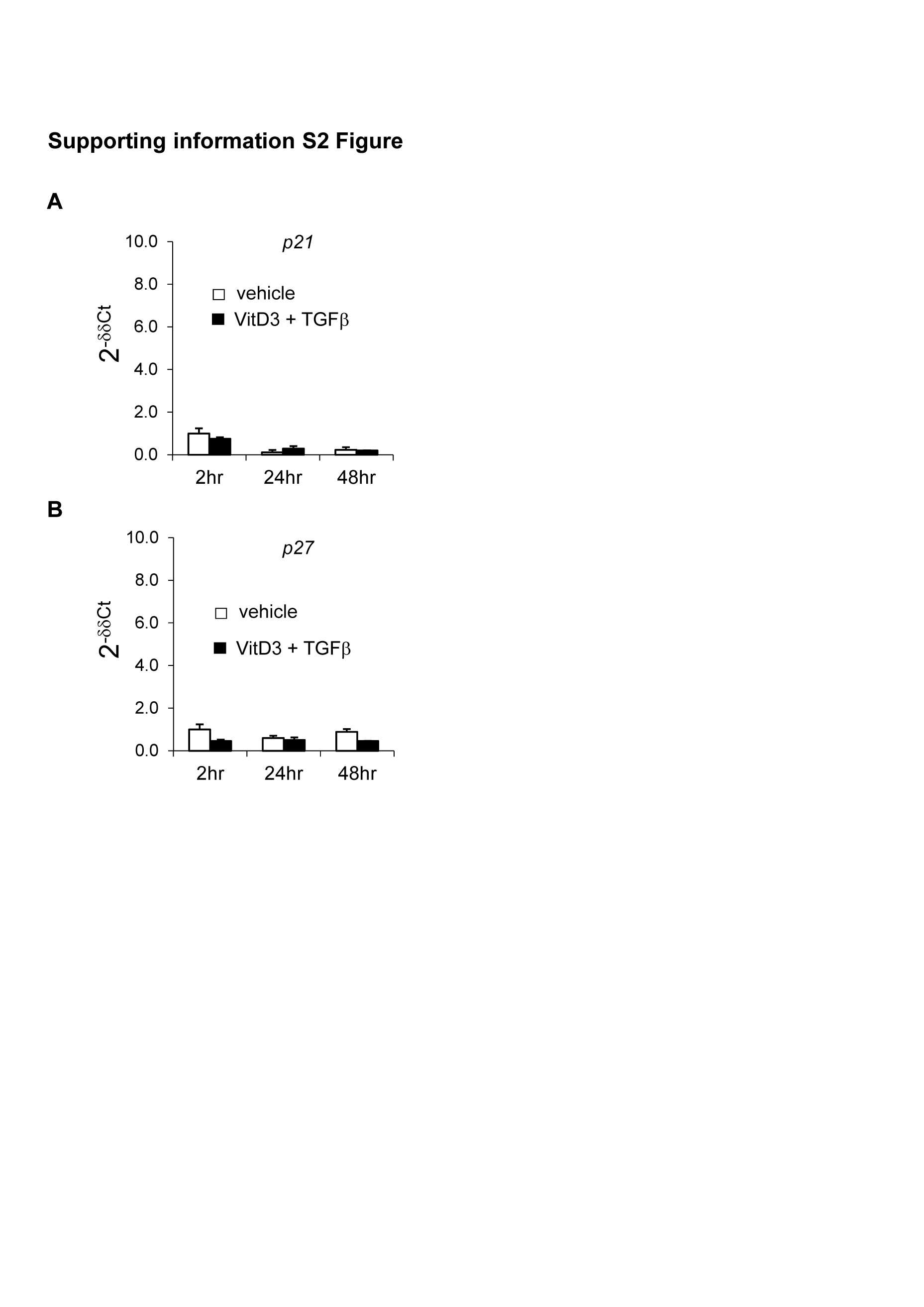

Supplement: S2 Fig — Real-time PCR analyses of p21 expression (A) and p27 expression (B) in MG-63, relative to 2hr vehicle sample. (TIF) [file pone.0128513.s003.tif]

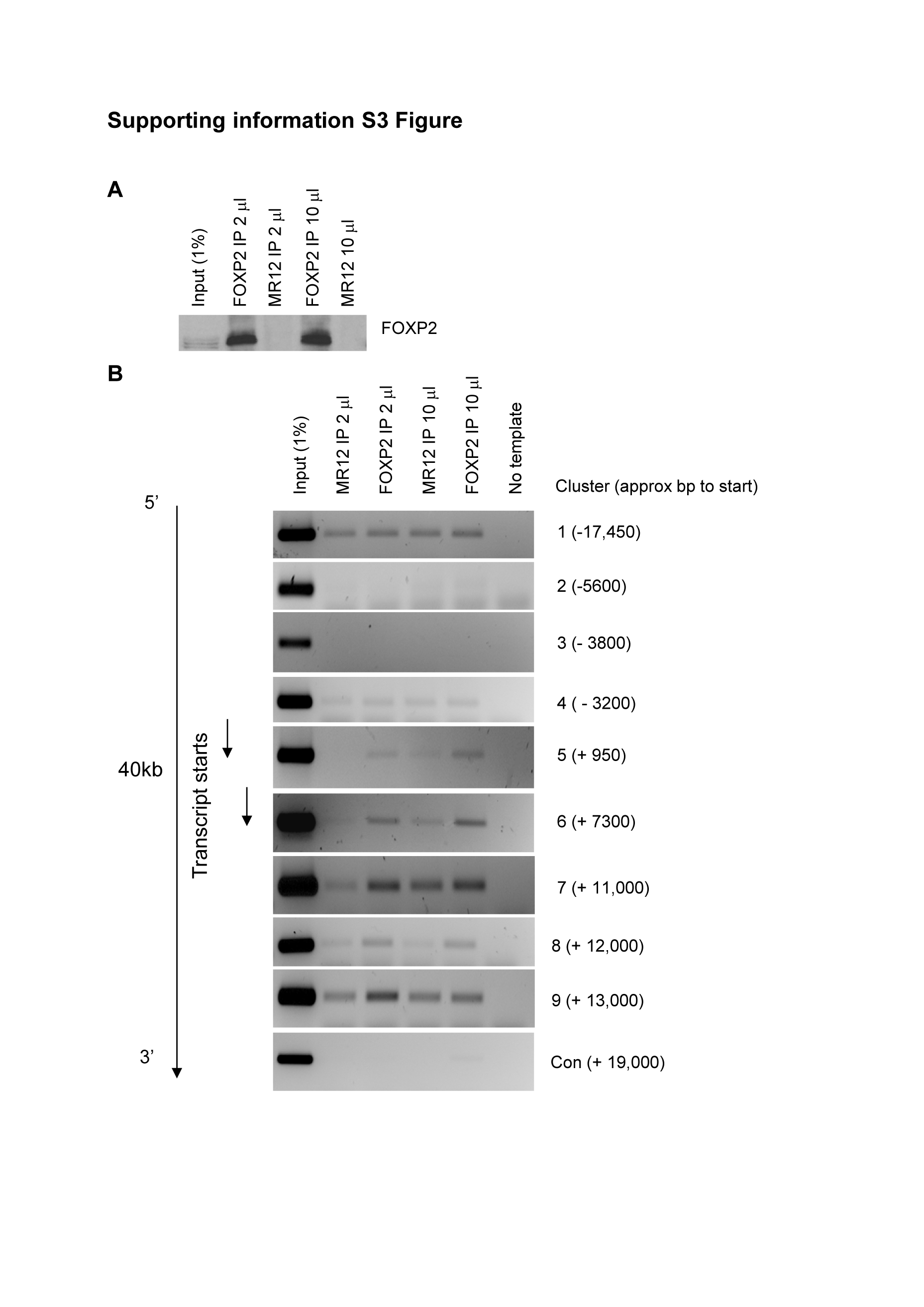

Supplement: S3 Fig — (A) Initially, confluent 143B cell lysates were immunoprecipitated with MR12 or 73A/8 FOXP2 antibodies as indicated, washed as per ChIP protocol and precipitated complexes analysed by immunoblot for presence of FOXP2 protein. (B) Co-precipitated chromatin from similar experiments was used as template for amplification of fragments of the human p21 WAF1/CIP locus. Site labelled ‘con’ was chosen as a negative control, being (within the 40kb analysed) the greatest distance away from predicted FOXP binding sites. Although some apparent FOXP2 binding at sites 5 to 8 was observed in the experiment shown it could not be replicated in a further five experiments. (TIF) [file pone.0128513.s004.tif]
